# Sleep Characteristics of Highly Trained Wheelchair Rugby Athletes With and Without a Cervical Spinal Cord Injury During the Competitive Season

**DOI:** 10.3389/fspor.2021.643233

**Published:** 2021-04-29

**Authors:** Conor J. Murphy, Iuliana Hartescu, Ifan E. Roberts, Christof A. Leicht, Vicky L. Goosey-Tolfrey

**Affiliations:** ^1^The Peter Harrison Centre for Disability Sport, School of Sport and Exercise Sciences, Loughborough University, Loughborough, United Kingdom; ^2^National Centre of Sport and Exercise Medicine (NCSEM), School of Sport and Exercise Sciences, Loughborough University, Loughborough, United Kingdom

**Keywords:** paralympics, disability, exercise recovery, sleep duration, sleep quality, sport, training camp

## Abstract

Sleep behaviors although significantly relevant to exercise recovery are poorly characterized in Para-sport athletes. Therefore, the main aims of this study were to describe sleep quality and quantity of highly trained wheelchair rugby (WR) athletes during the competitive season, and to investigate whether impairment type or attending a training camp influenced sleep outcomes. Eighteen male WR athletes (mean ± *SD*; age: 30 ± 5 years) with cervical spinal cord injuries (*n* = 11) (CSCI) and without (*n* = 7) (NON-SCI) wore an activity monitor over a 16-day period to objectively quantify sleep parameters, while the Pittsburgh Sleep Quality Index (PSQI) and nightly sleep diary entries were used as subjective means. A sub-sample of the athletes (*n* = 11) had their sleep monitored during a 3-night training camp to assess the impact of environmental change on sleep. Furthermore, as an additional exploratory measure core temperature was measured for a single night-time period using ingestible telemetry capsules. The athletes had total sleep times and sleep efficiency scores of 7.06 (1.30) h.min [median (interquartile range)] and 81 (9)%, respectively. Sleep onset latency and wake after sleep onset were 13 (24) min and 1.11 (0.45) h.min, respectively. No significant differences were found in objective sleep variables between the impairment groups despite the CSCI group being significantly more likely to report a poorer night's sleep (*p* = 0.04). Furthermore, attending the training camp caused a significant reduction in total sleep time for both groups [Δ38 ± 33 min; (95% CI: 18–60 min) *p* < 0.01]. This study highlights suboptimal sleep characteristics that are present in both CSCI and NON-SCI wheelchair athletes, as defined by the National Sleep Foundation. Although objective scores did not differ between groups, athletes with a CSCI rated their sleep worse. Furthermore, the disruption of sleep during training camp reflects an additional risk factor that is important to recognize for those working with wheelchair athletes.

## Introduction

Recovery is an essential component of training adaptation (Kellmann et al., 2018). The optimal balance between physical stress, resulting from training and competition, and periods of recovery is known to induce positive outcomes. However, when recovery is insufficient, overreaching, and subsequently overtraining may occur (Kreher and Schwartz, 2012). Acquiring sufficient sleep has been hypothesized as a one of the fundamental elements of the recovery process post-exercise.

The negative impact of elite sport on sleep quality has been proposed (Gupta et al., 2017). Competition, long-haul travel, and training load have been identified as pertinent risk factors that could disrupt sleep in athletes (Gupta et al., 2017). For example, Leeder et al. (2012) demonstrated that elite athletes monitored during the competitive season showed signs of diminished sleep quality compared to non-athletic populations. Studies that compare rest to training days have reported earlier wake times and reduced total sleep time on training days (Sargent et al., 2014a,b). To further compound this, sports that schedule training earlier in the day are associated with lower total sleep times and higher levels of pre-training fatigue (Sargent et al., 2014b). Moreover, it is acknowledged that periods of increased training load may have an influence on both sleep quality and quantity (Kölling et al., 2016; Killer et al., 2017). Therefore, events such as training camps have the potential to disturb sleep due to a combination of factors including early morning training, increased training volume and/or intensity, and challenges associated with sleeping away from home (i.e., shared rooms, single beds, etc.).

The aforementioned studies have all been conducted with able-bodied sports persons. Paralympic athletes may face additional factors that interfere with their ability to achieve sufficient sleep due to impairment specific problems. For example, individuals with a spinal cord injury (SCI) commonly report disruptions to their sleep due to muscle spasms, paraesthesia, voiding, and uncomfortable temperature sensations (Biering-Sørensen and Biering-Sørensen, 2001). According to one large survey up to 78% of individuals with an amputation still experience phantom pain, a disorder that can persist during sleep (Sherman et al., 1984), while individuals with achondroplasia are prone to sleep disordered breathing due to facial hypoplasia (Pauli, 2019). Furthermore, core temperatures (T_core_) rhythms, which are linked to the initiation of sleep, may be altered in particular impairment groups, such as individuals with a SCI at the cervical level (CSCI) (Thijssen et al., 2011), however, more work is required to confirm this.

Although research specific to Para-sport is limited, a small number of studies have been conducted. The two qualitative studies that exist highlight that up to 70 and 38% of para-athletes prior to the Beijing and London Paralympic games demonstrated poor subjective sleep quality, respectively (Silva et al., 2012; Rodrigues et al., 2015). The disparity between these studies likely relates to the different types of sports and impairment groups included in each study. However, recently published research using actigraphy demonstrated concerningly low total sleep times (<5.30 h.min) in a small group of wheelchair rugby (WR) athletes pre and in-season (Sanz-Milone et al., 2020). Nonetheless, the study was limited to six participants, all of which were athletes with a CSCI. Wheelchair rugby includes individuals with a diverse range of impairments and functional classifications. Therefore, the description of sleep-wake patterns in athletes without a SCI (NON-SCI) is also of importance, as they may differ, particularly from a thermoregulatory perspective (Thijssen et al., 2011). Furthermore, in the study by Sanz-Milone et al. (2020) training was restricted to 3 days per week, thus, the results may not fully reflect in-season sleep-wake patterns when training is not controlled.

Therefore, the main aims of this study were to (1) describe sleep quality and quantity of highly trained WR athletes, during the competitive season, using objective and subjective measures; (2) compare sleep between athletes with a CSCI and NON-SCI; and (3) compare sleep patterns when at home and during a training camp. Furthermore, as an exploratory analysis on a small sub-sample of data, present night-time T_core_ within CSCI and NON-SCI. We hypothesized that (1) the whole group would have suboptimal sleep characteristics (2) no differences in outcomes would be found between athletes with a CSCI and NON-SCI (3) sleep patterns would significantly change during the training camp period.

## Materials and Methods

### Subjects

From a pool of 40 athletes, 18 highly trained male WR players volunteered to participate (mean ± *SD*; age: 30 ± 5 years). Eleven had tetraplegia (one C4/5; two C5; two C5/6; three C6; two C6/7; one C7), three had multiple limb amputations, one was affected by critical illness polyneuropathy, one by Roberts syndrome, one by osteogenesis imperfecta, and one by cerebral palsy. All seven WR Paralympic classifications were represented (from 0.5 to 3.5) (Tweedy and Diaper, 2010). Highly trained was classed as any athlete involved in a national programme from a top five WR nation. Participants were above 18 years of age, not returning from transmeridian travel (+2 timezones) in the two prior weeks, and were otherwise healthy and independent of any form of sleep medication. All participants were aware of the purpose of the study and provided written informed consent to participate. The study was approved by local ethical advisory committee in line with the Helsinki Declaration (R18-P135).

### Study Design

**Figure 1:**
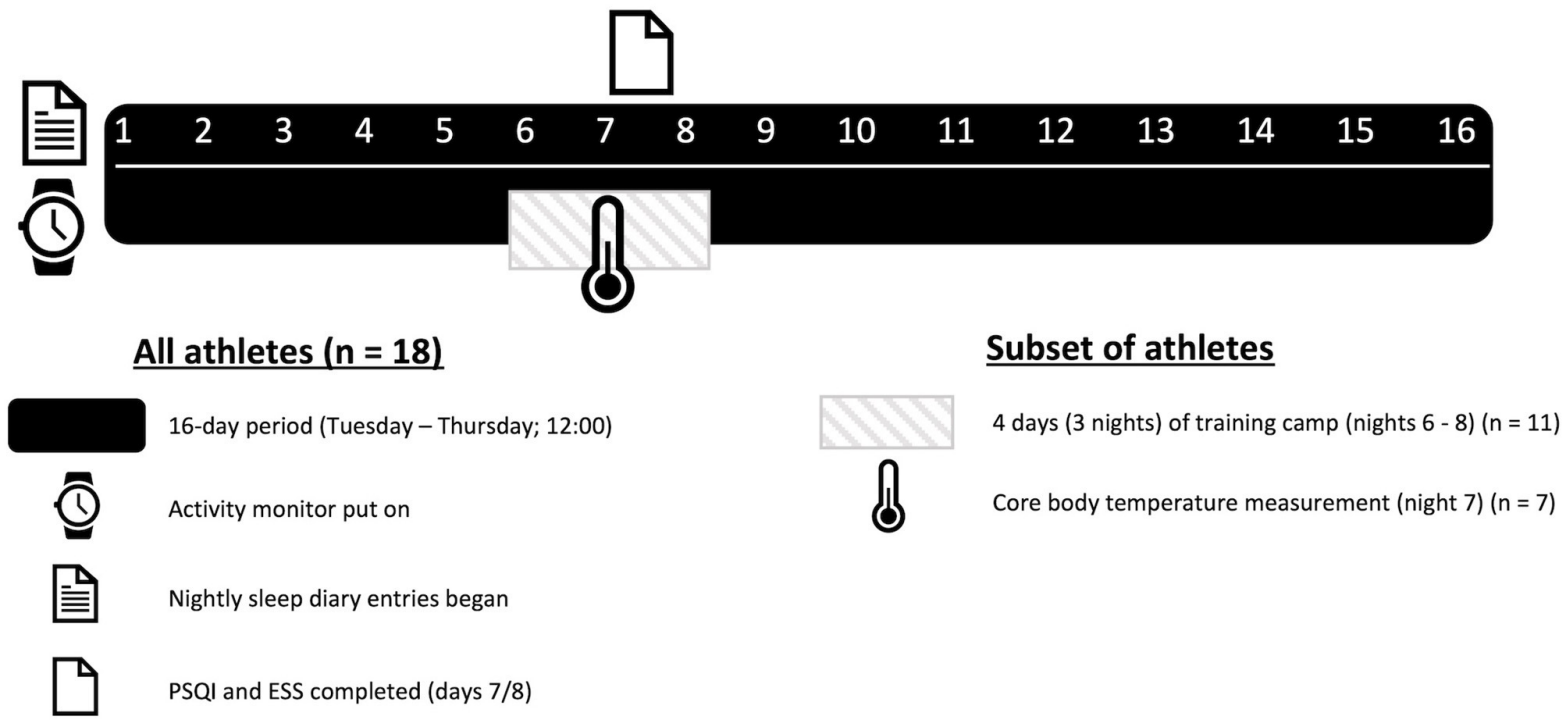
Study design.

Eighteen participants wore an activity monitor wristwatch on their non-dominant arm and completed a daily sleep diary for a 16-day period during the competitive WR season. Within the 16-day sleep monitoring period, a sub-group of 11 participants were assessed during a 3-night training camp at an affiliated training site, which the players resided at. The camps are regularly organized to provide an opportunity for increased training during the competitive season. As an additional exploratory measure, during the training camp, seven participants ingested a telemetry capsule to allow the collection of T_core_ during a single night-time period (night 2 of camp). Figure 1 shows the study design.

### Actigraphy

Participants were instructed to wear an activity monitor (Motionwatch 8, CamNtech, Cambridgeshire, UK) continuously for the duration of the study. There is support for actigraphy as a valid method for monitoring sleep in populations with a SCI from C5–C7 (Spivak et al., 2007). A 16-day period was selected as to permit sufficient time to establish habitual sleep patterns (Van Someren, 2007). If the activity monitor was removed the participant was asked to record the time and date in the sleep diary. Sleep and wake cycles were identified using the event marker button on the activity monitor, where participants indicated “bed time” (attempt made to sleep) and “lights on time” (finished attempting to sleep). If participants failed to press the event marker button (<10% of data), cycles were identified using participants' sleep diaries and the software's automatic scoring algorithm. Actigraphy data were sampled at 30-s epochs, and analyzed using MotionWare software (version 1.2.28, CamNtech, Ltd.). The movement threshold was set at high sensitivity (20 cpm). Sleep variables analyzed included; bed time (BT), wake time (WT), time in bed (TIB), total sleep time (TST), sleep onset latency (SOL), sleep efficiency (SE) defined as total sleep time expressed as a percentage of time between “bed time” and “lights on time,” wake after sleep onset (WASO) being the total wake time between sleep onset and “wake time,” and sleep fragmentation (SF) as the frequency of movement during sleep. Non-parametric circadian rhythm analysis (NPCRA) (Van Someren et al., 1999) was used to quantify circadian stability. Non-parametric circadian rhythm analysis uses the activity counts per epoch as the basis for calculations (counts are the sum of the 1 s peak values over the epoch): inter-daily stability (IS) of activity, intra-daily variability (IV) of activity, least active 5 h (L5) start time, most active 10 h (M10) start time, and relative amplitude (RA) of peak and nadir activity were measured and reported for the purpose of this study, see the above paper for full descriptions.

### Sleep Questionnaires

Participants were asked to complete a short sleep diary each day during the study period. Subjective sleep quality ratings (very good, good, fair, poor, very poor) for each night were obtained from the scale within the diary. Napping information was reported as a diary entry. The participants also completed the Pittsburgh Sleep Quality Index (PSQI) (Buysse et al., 1989). The PSQI consists of 21 items that subjectively evaluate sleep quality over the previous month. Scores across seven components are added to yield one “global” score, with a range of 0–21 points, “0” indicating no difficulty and “21” indicating severe difficulties in all components. A global PSQI cut-off score >5 is a commonly used marker of poor sleep (Buysse et al., 1989). Finally, the Epworth Sleepiness Scale (ESS) was completed to determine levels of sleepiness among the participants. Daytime sleepiness was defined as normal (0–5), limit (6–10), mild (11–12), moderate (13–15), and severe (above 16) (Johns, 1991). Both the PSQI and ESS were completed during the beginning of the second week (days 7 and 8) of the study.

### Training Camp

The 11 athletes arrived at the training camp prior to 1 pm before the first training session that day. The training camp consisted of two full (~6 h active time) and two half training days (~3 and 2.5 h active time, arrival and departure days, respectively). Thus, the volume of exercise consisted of 17–18 h of active training over a 4-day period. The types of training reflected those commonly performed during each athlete's normal training sessions including a combination of warm-ups, drills, tactical plays, intensity manipulated games, match simulations and cool-downs. Therefore, the type of training was similar to that seen outside of camp, but the volume of work was increased, as these athletes typically complete two to three WR sessions per week (2–3 h session) interspersed with strength and conditioning work. Weekly court-based training volumes were reported to be on average below 10 h by the performance support team. Training start times were 3 p.m. on day 1 and 9:30 a.m. for all other days. Training end times were 6:15, 5:15, 5:15, and 12 p.m. for days 1–4, respectively. The sleep environment consisted of shared rooms with single beds. Furthermore, the participants were free to self-select their bed-time routine, clothing, and room temperature to mimic a normal training camp environment. Each bedroom included an inbuilt thermostat.

### Core Temperature

Gastrointestinal temperature, as an indicator of T_core_, was measured using an ingestible telemetry capsule (HQ Inc., Palmetto, Florida, USA) (Bongers et al., 2018; Au et al., 2019). Participants swallowed the capsules the night before the measurements were taken to minimize the influence of fluid or food on temperature readings, in line with previous recommendations (Byrne and Lim, 2007). T_core_ was measured every 20 s using Cortemp data monitors.

### Statistical Analysis

All T_core_ data were firstly filtered to remove any outliers within the data set using, MathWorks software (R2015b, Inc., London, UK). Outliers were identified as data points with >0.30°C difference between the measurements taken immediately before, and those taken after the data point in question. T_core_ data were averaged over 15 min time periods and parameters were calculated from the night-time period (BT to WT).

Normality of data for each night were checked by Shapiro–Wilk tests. Measures of centrality and spread are presented as mean ± standard deviation (95% confidence interval of the mean [CI]) for normally distributed data and median (interquartile range) for non-normally distributed data. However, for comparative purposes, group data for each objective sleep parameter are reported as median (interquartile range) even if one subset was normally distributed. The coefficient of variation (CV; CV = [*SD*/mean] × 100) for TST and SE across the study period was calculated intra-individually. BT, WT, TIB, TST, SOL, WASO, SE, and SF were compared between impairments categories (CSCI vs. NON-SCI) using a linear mixed model analysis. The nights of the study period (i.e., 1–16), the impairment categories (CSCI vs. NON-SCI), and their interaction were included as fixed effects, participants as the random effect, and the environment (home or training camp) as a covariate. The NPRCA parameters and sleep questionnaire scores (PSQI and ESS) were compared between impairments categories (CSCI vs. NON-SCI) using an independent samples *t*-test. A chi-square analysis was used to determine if any association existed between impairment category and the prevalence of good (good and very good rating) vs. bad (poor and very poor rating) nights of sleep.

As a sub-analysis, differences between sleep activity patterns while at home and during training camp were evaluated using a within-subjects linear mixed model analysis (for sub-sample, *n* = 11). The model included fixed effects of environment (home vs. training camp) and the random effect of participant. Furthermore, Bonferroni-corrected pairwise comparisons were used to show the day-to-day differences across the study period for the sub-sample that attended training camp. For each linear mixed analysis, scaled identity was used for the variance-covariance structure model, which assumes constant variance and no correlation between data, chosen via the smallest Akaike Information Criterion (AIC) method.

Differences between CSCI and NON-SCI groups in T_core_ parameters were computed as descriptives. Statistical analyses were performed using the Statistical Package for the Social Sciences (Version 26.0; SPSS, Inc., Chicago, USA). The level of significance was set at *p* < 0.05.

## Results

### Missing Data

A sub-sample (*n* = 3) of athletes did not receive their activity monitors before the commencement of the study [1 day (*n* = 2); 2 days (*n* = 1)] resulting in a small number of missing data points [each sleep variable: valid cases, *n* = 284 (98.6%), and missing cases, *n* = 4 (1.4%); sleep diary entries: valid cases, *n* = 279 (96.8%), and missing cases, *n* = 9 (3.1%)].

### Sixteen-Day Overall

**Figure 2:**
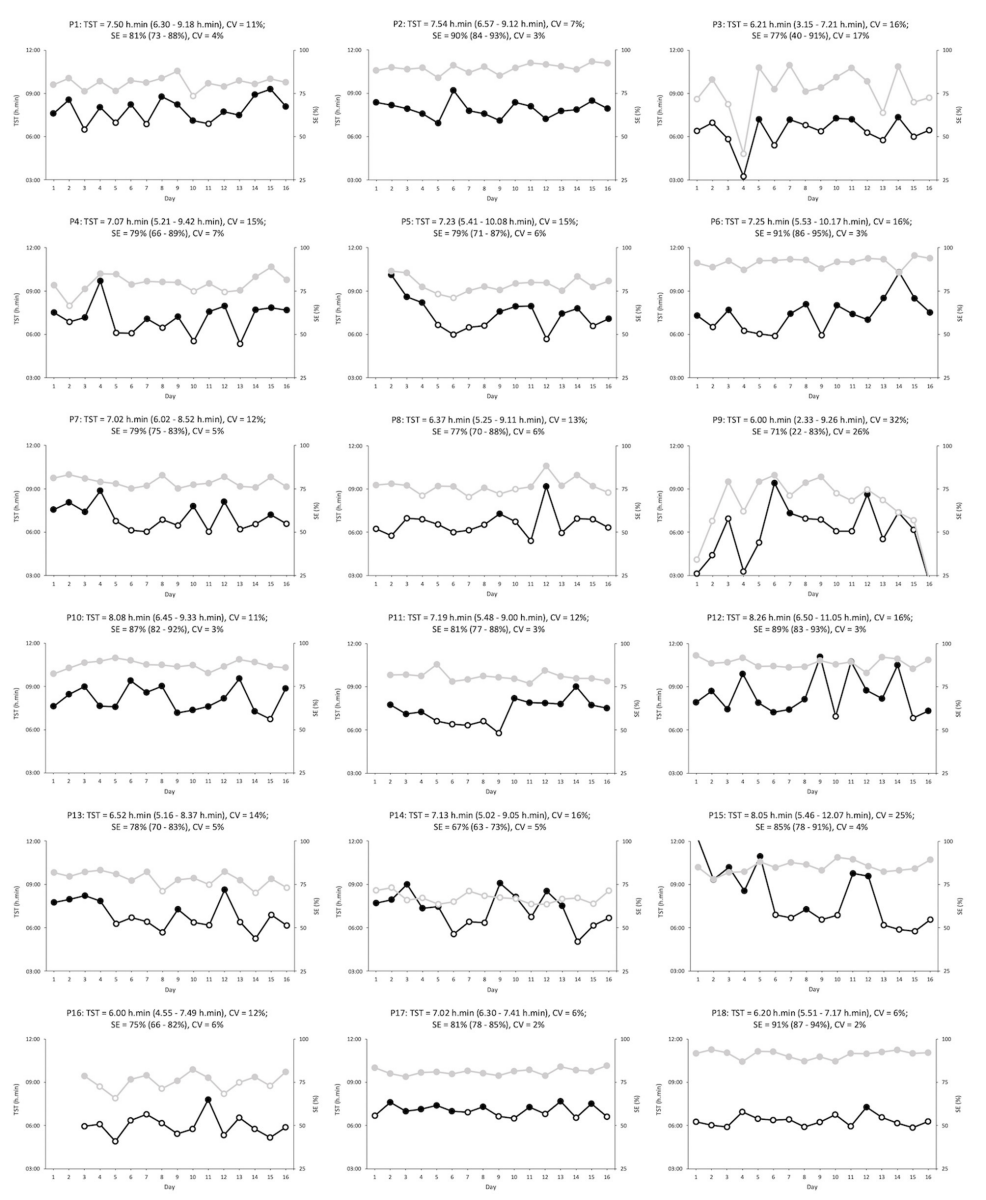
Total sleep time (black) and sleep efficiency (gray) for each participant (*n* = 18) over the 16-day period. Open symbols indicate total sleep times below 7.00 h.min or sleep efficiencies below 75%. Each heading contains the participant number; the average (with minimum and maximum values), and the variability, for both total sleep time and sleep efficiency. P9 (night 16) = total sleep time of 2.33 h.min and sleep efficiency of 22%, and P15 (night 1) = total sleep time of 12.07 h.min. P, participant; CV, coefficient of variation; TST, total sleep time; SE, sleep efficiency.

Overall data for each sleep parameter are recorded in Table 1. To note, individual data showed 11% of athletes (2/18) had a SE score below 75%, one-third (6/18) had a TST of <7.00 h.min and 89% (16/18) had a WASO value above 41 min. Inter-daily stability, as an indicator of inter diurnal rhythm, was 0.35 ± 0.14 (0.29–0.42) AU. In addition, IV and RA were 0.66 ± 0.16 (0.59–0.74) and 0.92 ± 0.03 (0.91–0.93) AU, respectively. The L5 start hour was 01:18 ± 00:46 (00:32–01:39) hh:mm and the M10 start hour was 09:03 ± 01:23 (07:40–09:41) hh:mm. Over the study period, subjective sleep quality, as assessed using the daily sleep diary, was rated as very poor or poor 16% of times, fair 30% of times, and good or very good 54% of times. Napping was used at least once by 50% of the athletes as indicated by sleep diary entries (*n* = 9). However, those that napped only did so 3 ± 2 (2–4) times over the course of the study, which ranged from 15 to 120 min. The overall mean PSQI score was 5.61 ± 3.13 (4.06–7.17). The most commonly reported causes for trouble sleeping in the PSQI were “cannot get to sleep within 30 minutes” (*n* = 13), “wake up in the middle of the night or early morning” (*n* = 13), “feel too hot or feel too cold” (*n* = 11), “have to use the bathroom” (*n* = 6) and “have pain” (*n* = 5). The mean ESS score was within the daytime sleepiness limit category (6–10). Overall values for both the PSQI and ESS are presented in Table 1. Furthermore, individual data for TST and SE across each night of the study are displayed in Figure 2.

**Table 1:** Objective sleep actigraphy and subjective questionnaire data comparing CSCI vs. NON-SCI.

**Group**	***n***	**Bed time (hh:mm)**	**Wake time (hh:mm)**	**Time in bed (h.min)**	**Total sleep time (h.min)**	**Sleep onset latency (min)**	**Wake after sleep onset (h.min)**	**Sleep efficiency (%)**	**Sleep fragmentation (AU)**	**PSQI (0–21)**	**ESS (0–24)**
Overall	18	23:12 (1:23)	07:58 (1:54)	8.42 (1.41)	7.06 (1.30)	13 (24)	1.11 (0.45)	81 (11)	33 (24)	5.61 ± 3.13 (4.06–7.17)	5.78 ± 3.12 (4.23–7.33)
CSCI	11	23:09 (1:19)	08:11 (1:34)	8.52 (1.32)	7.24 (1.27)	10 (22)	1.07 (0.46)	82 (11)	35 (22)	5.91 ± 3.48 (3.57–8.25)	5.55. ± 3.70 (3.06–8.03)
NON-SCI	7	23:23 (1:18)	07:18 (2:04)	8.27 (1.53)	6.37 (1.09)	17 (25)	1.19 (0.44)	79 (11)	29 (25)	5.14 ± 2.67 (2.67–7.62)	6.14 ± 2.12 (4.19–8.10)

*Values are mean ± standard deviation (95% confidence interval of the mean) and median (interquartile range). CSCI, cervical spinal cord injury; NON-SCI, without a spinal cord injury; PSQI, Pittsburgh Sleep Quality Index; ESS, Epworth Sleepiness Scale*.

Sleep variables across each different impairment category can be seen in Table 1. No significant fixed effects were found across group (CSCI vs. NON-SCI) in any of the measured sleep parameters (*p* > 0.05). BT (*p* < 0.01), and WT (*P* < 0.01) were significantly influenced by the night (fixed effect) that was measured, while group differences between nights (night/group interaction) was significant across WT (*p* = 0.04), TIB (*p* = 0.04), TST (*p* = 0.02), and WASO (*p* = 0.02). Furthermore, no significant differences were found between groups in the measured NPCRA variables (*p* > 0.05). Chi-square analysis revealed that the likelihood to experience either good or bad sleep was affected by group (*p* = 0.04). Very poor or poor nights were experienced 19% of times and 10% of times by the CSCI and NON-SCI groups, respectively. Similarly, the NON-SCI group reported good and very good nights 9% more than the CSCI group. Both the PSQI and ESS scores did not differ between groups (*p* = 0.61 and *p* = 0.70, respectively).

### Training Camp

**Figure 3:**
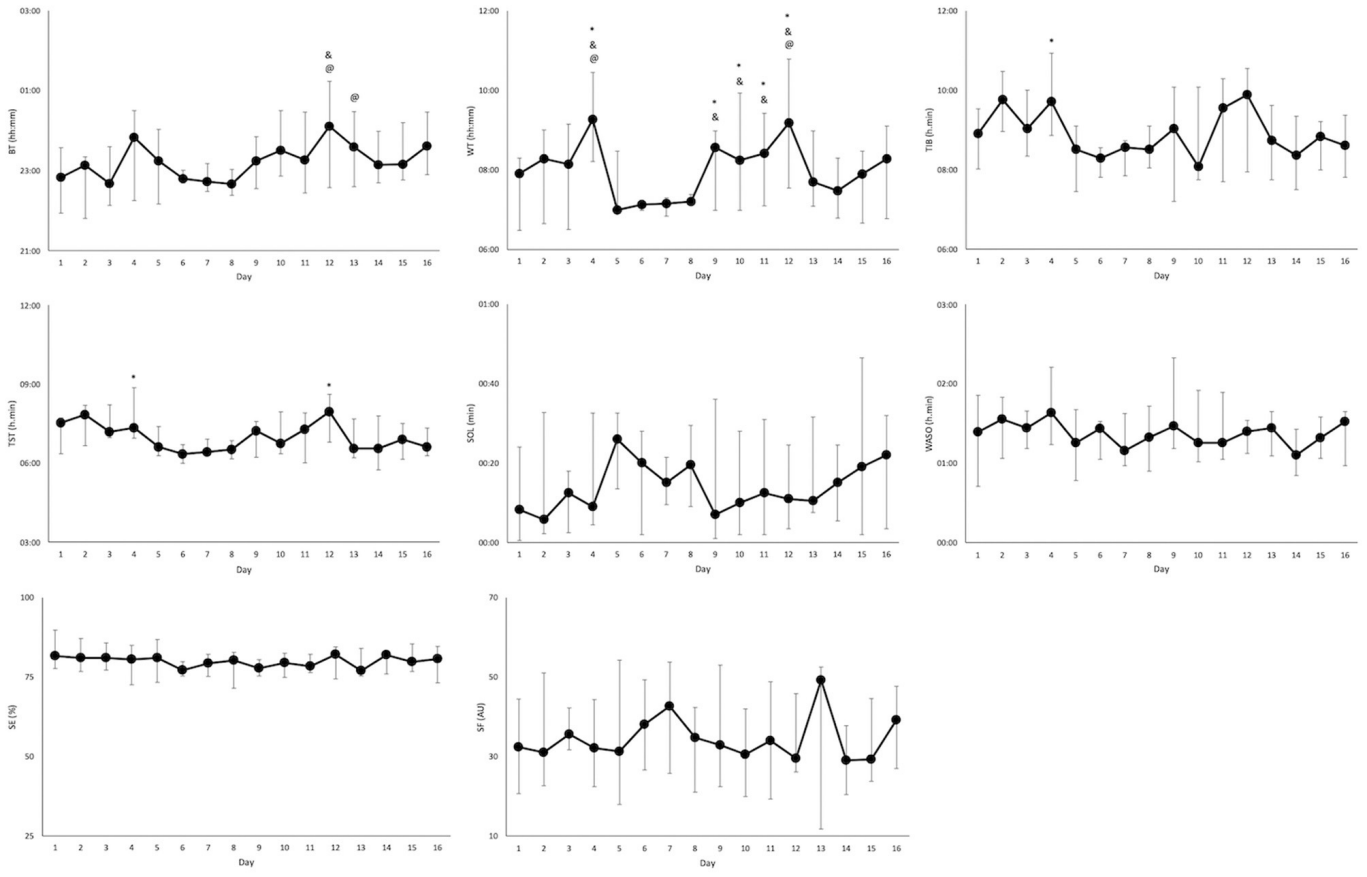
Sleep parameters (median and interquartile range) over the 16-day period for the athletes that attended training camp (*n* = 11). Significant differences from night 1 of camp (night 6), night 2 of camp (night 7), and night 3 of camp (night 8) are indicated by *, &, and @, respectively. BT, bed time; WT, wake time; TIB, time in bed; TST, total sleep time; SOL, sleep onset latency; SE, sleep efficiency; WASO, wake after sleep onset; SF, sleep fragmentation.

Sleep data from 11 of the players (5 CSCI, and 6 NON-SCI) were analyzed during a training camp (Table 2). Total sleep time was lower during the training camp compared to at home [Δ38 ± 33 min; (18–60 min) *p* < 0.01]. There was also an earlier BT (*p* = 0.02) and WT (*p* < 0.01) at camp, but a reduction in TIB (*p* = 0.01). Sleep parameter changes across the study period for the athletes that attended the training camp are displayed in Figure 3.

**Table 2:** Sleep actigraphy data comparing sleeping at home vs. during the training camp.

**Environment**	***n***	**Bed time (hh:mm)**	**Wake time (hh:mm)**	**Time in bed (h.min)**	**Total sleep time (h.min)**	**Sleep onset latency (min)**	**Wake after sleep onset (h.min)**	**Sleep efficiency (%)**	**Sleep fragmentation (AU)**
Home (13-night period)	11	23:17 (1:31)*^*a*^*	08:13 (2:07)*^*a*^*	8.55 (2.00)*^*a*^*	7.06 (1.29)*^*a*^*	13 (26)	1.25 (0.36)	80 (8)	33 (23)
Training camp (3-night period)	11	22:45 (0:30)*^*a*^*	07:09 (0:17)*^*a*^*	8.25 (0.41)^a^	6.25 (0.40)*^*a*^*	19 (17)	1.19 (0.36)	79 (7)	36 (25)

*Values are median (interquartile range). Mean values with the superscript a signify significant differences between home and training camp (p < 0.05)*.

**Figure 4:**
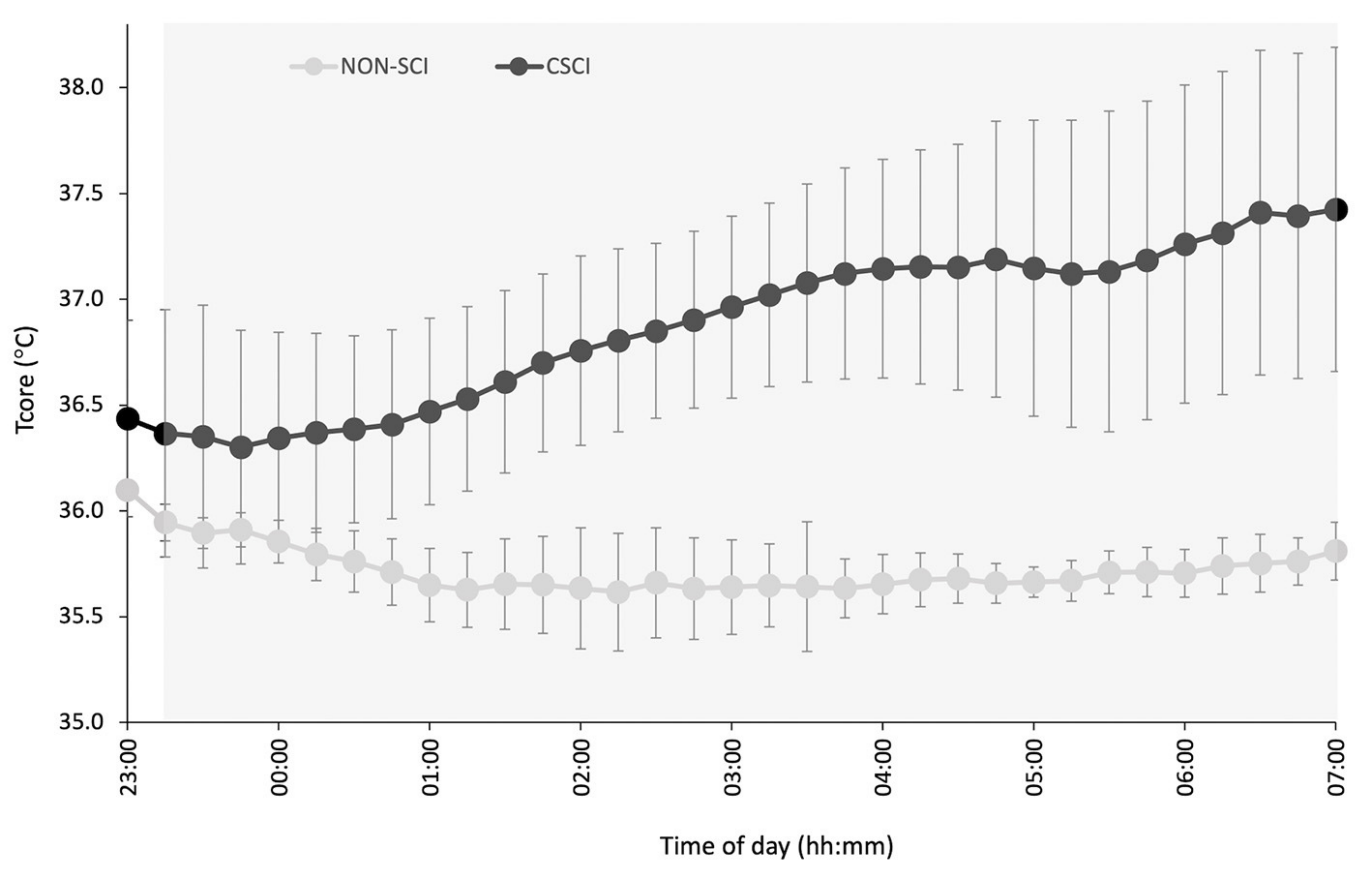
Night-time T_core_ in participants with a cervical spinal cord injury (CSCI; *n* = 3) and without a spinal cord injury (NON-SCI; *n* = 4). The gray region represents the average time of sleep for all subjects. Data are presented as a mean over a 15 min period, whereas error bars represent SE.

T_core_ during the night-time period is displayed in Figure 4, furthermore, descriptive T_core_ variables are reported in Table 3.

**Table 3:** Night-time parameters of T_core_.

**Variable**	**CSCI (*n* = 3)**	**NON-SCI (*n* = 4)**
Mean T_core_ during sleep (°C)	36.9 ± 1.0 (35.8–38.0)	35.7 ± 0.3 (35.4–36.1)
Minimum T_core_ value (°C)	36.3 ± 0.8 (35.4–37.2)	35.6 ± 0.4 (35.2–36.0)
Minimum T_core_ value time (hh:mm)	23:30 ± 00:54 (22:28–00:31)	03:41 ± 01:44 (01:59–05:22)
h before wake time (h.min)	7.35 ± 0.38 (6.52–8.18)	3.15 ± 1.57 (1.20–5.10)
h after bed time (h.min)	0.50 ± 0.48 (0.00–1.44)	4.53 ± 1.27 (2.28–5.18)

*Values are mean ± standard deviation (95% confidence interval of the mean). CSCI, cervical spinal cord injury; NON-SCI, without a spinal cord injury; h, hours; T_core_, core temperature*.

## Discussion

This study evaluated sleep quality and quantity both objectively and subjectively in a highly trained cohort of male WR athletes including both athletes with a CSCI and NON-SCI. As a whole, the athletes were on the borderline between moderate to poor quality sleep reflected by suboptimal sleep characteristics (Hirshkowitz et al., 2015; Ohayon et al., 2017), and although no differences in objective sleep were found between CSCI and NON-SCI groups, athletes with a CSCI subjectively reported worse sleep. Furthermore, poor sleep quality was further exacerbated during a training camp period. Our hypotheses that suboptimal sleep characteristics would be present in both groups was confirmed, that no differences would exist between groups was rejected, and that sleep would be influenced by attending a training camp was accepted. Furthermore, our exploratory analysis of the small sub-sample of data revealed earlier timing of the night-time T_core_ minimum in the CSCI compared to the NON-SCI group.

The group SE score (~81%) gathered over the 16-day period, although above the value considered as inappropriate (<75%), falls within a suboptimal zone as only values above 84% are agreed upon as appropriate for good sleep quality (Ohayon et al., 2017). Furthermore, a marker that suggested poor sleep quality in this group of athletes was the high WASO score (1.11 h.min) which well surpassed the inappropriate threshold for young adults and adults (>41 min) and even for older (>65 years) adults (+ 61 min) (Bongers et al., 2018). This is a notable finding, emphasized by the fact that 16/18 of the athletes had overall WASO scores above 41 min. Importantly, with regard to SE, studies show that even values within the recommended range are associated with decreased cognitive performance amongst athletes (Akazawa et al., 2019), and that each 5% increase in SE is accompanied by a significant increase in reported sleep quality (Akerstedt et al., 1994). From a long-term health perspective, there are established relationships between inadequate sleep efficiency values and excess mortality (Kripke et al., 2011). Furthermore, although the TST was within the recommended duration (7–9 h), it only surpassed this recommendation by ~6 min (Hirshkowitz et al., 2015), leaving it vulnerable to incidental sleep duration disruptions. The WR athletes in the present study had contrasting data compared to WR athletes with a CSCI in the recent study by Sanz-Milone et al. (2020). The athletes in this previous study had optimal SE scores (>85%) (Ohayon et al., 2017), but a very low TST of <5.30 h.min (Hirshkowitz et al., 2015). Thus, the increased SE for the group of athletes assessed by Sanz-Milone et al. (2020) may reflect sleep deprivation in their sample. Nonetheless, it is noteworthy to mention individual data, for example, one-third (*n* = 6) of the athletes in the present study also had overall TST values below 7.00 h.min. This is relevant as consecutive days of <7 h sleep has been shown to increase the risk of developing an upper respiratory tract infection (Cohen et al., 2009), and to result in cumulative deficits in cognitive performance (Van Dongen et al., 2003).

Individual information relating to the range and day-day variability of TST and SE is further highlighted in Figure 2 and provides important unmasked information. Although one-third of the athletes during the study period had overall TST values below 7.00 h.min it is apparent from Figure 2, that all, but one athlete, had at least one night below this recommended value (Hirshkowitz et al., 2015). Concerningly, 12 of the athletes slept <7.00 h.min for more than one-third (>five) of the nights. It is possible that some of the athletes may be classed as “short sleepers,” and therefore, require less total sleep for daily living. The day-day variability presented may provide an insight into this. For example, TST variability for both P17 and P18 was low suggesting that they regularly obtain consistent sleep durations without disruptions or the need for rebound sleep periods afterwards. On the other hand, participants, such as P9 and P15, had large variability in TST which could indicate the presence of inappropriate sleep followed by rebound sleep during the subsequent nights. Unfortunately, chronotype information may have helped further characterize “short” and “long” sleepers but this information was not recorded for this study.

A comparison of groups showed no significant differences in the objective sleep variables measured. Although a trend suggested athletes with a CSCI had longer and more efficient sleep, these differences did not reach statistical significance. The CSCI TST score was higher (7.24 [1.27] vs. 6.37 [1.09] h.min) than the NON-SCI group throughout the study. Yet interestingly, the CSCI group reported poorer quality sleep (very poor and poor) more often than the NON-SCI group who rated a higher proportion of good and very good nights. A mismatch in subjective and objective markers of sleep is a common trait in insomnia but has also been shown in male athletes (Schaal et al., 2011). This contradiction highlights that the perception of sleep may not always represent objectively obtained information (Edinger et al., 2000; Schaal et al., 2011). Common troubles reported in the PSQI, such as thermal discomfort (*n* = 11) and pain (*n* = 5), could negatively influence the perception of sleep, yet the objective scores may not reflect this. Furthermore, many athletes reported “cannot get to sleep within 30 minutes” (*n* = 13) in the PSQI. Although the objective SOL scores are within a normal range (Ohayon et al., 2017), the large dispersion may explain this intermittent difficultly to initiate sleep. Speculatively, in athletes with a CSCI this could be related to physiological disruptions related to melatonin and core temperature rhythms both of which are linked to the initiation of sleep (Thijssen et al., 2011; Whelan et al., 2018).

Training camps are environments that may present challenges to attain appropriate sleep, therefore, a comparison was made in-season between sleeping at home and during a 3-night training camp. Most notably, a significant decrease in TST was observed, particularly during the first night of camp. This is likely due to the earlier start times associated with being at a training camp. Wake times during the training camp were significantly earlier than at home, reflecting studies whereby TST was reduced with early morning training (Sargent et al., 2014a; Kölling et al., 2016). However, the athletes in this study had a wake time of 07:09 (0:17) hh:mm at camp which is later than many sports which begin training at this time (Sargent et al., 2014b; Kölling et al., 2016). In addition, trying to compensate for sleep loss with earlier bedtimes may prove challenging as bedtime will then closely coincide with the wake maintenance zone, thus, impairing sleep initiation. Thus, focusing on a means to improve sleep consolidation and maintenance for this group is a priority, as an improved SE of 85%, if combined with the TIB achieved during camp, would have resulted in over 7 h of sleep. For example, appropriate bed time temperatures (Harding et al., 2019), cognitive behavioral therapy (Haynes et al., 2018), or sleep hygiene techniques (Vitale et al., 2019) are options that may be helpful. Although decrements in performance are less likely with short periods of sleep loss, training camps may span 1 week or more, therefore, the cumulation of partial sleep loss would require adequate planning and strategies to ensure recovery needs are met.

This study also measured night-time T_core_ for a sub-sample of the athletes and may provide another explanation for the subjectively reported poorer sleep from the athletes with a CSCI. Albeit a small sub-sample of the cohort, in the exploratory descriptive results presented all the athletes with a CSCI (*n* = 3) reached a minimum in T_core_ earlier than the NON-SCI group, and was succeeded by a steady increase in T_core_. Thijssen et al. (2011) observed a similar response in T_core_ speculating that the T_core_ circadian variation could be a pathophysiological mechanism that contributes to the high incidence of sleep disorders in individuals with a CSCI. Moreover, the circadian modulation of sleep stages has been postulated. For example, the propensity to enter rapid eye movement (REM) sleep is at its maximum shortly after the T_core_ minimum, while slow wave activity is highest at the circadian maximum of T_core_ (Dijk and Czeisler, 1995)_._ Thus, an advanced T_core_ rhythm may have implications on sleep architecture. Both REM and slow wave sleep have their own yet important roles for athletic recovery. Slow wave sleep is required for physiological recovery following exercise and increases following intense exercise (Shapiro et al., 1981). Furthermore, an early T_core_ minimum could advance morning wake zones which would increase the occurrence of arousals and interfere with the athletes ability to maintain sleep (Lack et al., 2008). Interestingly, WASO was inappropriate for both groups (good: <20 min) (Ohayon et al., 2017) so it is difficult to discern whether an impairment in T_core_ is responsible for the increased night-time wakefulness in the CSCI group. Polysomnography would be required to understand whether an alteration in thermoregulatory circadian rhythms do have an impact on sleep architecture in athletes with a CSCI.

## Limitations and Strengths

The present study is not without its limitations. A larger sample size with additional participants in each impairment group would have increased statistical power. Nonetheless, the study is focused on a very specific and small population group, for which little data exists. Furthermore, each athlete was assessed over the same 16-day period which allows more robust comparisons between groups and individuals. It is now well-established that sleep needs may differ, with individuals being characterized as short or long sleepers. Unfortunately, our data did not allow a distinction between both types of sleepers, and although chronotype information may have assisted, this measure was not recorded. The use of a gold-standard method, such as polysomnography, would have increased the validity of the data when compared with the chosen actigraphy approach, however, this was not a viable option in a group of athletes over a continuous period of 16-days. In addition, the sample size (*n* = 7) for the measurement of T_core_ was again small and was therefore only analyzed as an additional exploratory measure. Notably, however, it did display similar trends to those reported previously in recreational persons with a SCI, providing justification for further work (Thijssen et al., 2011). Lastly, three participants had some missing data, but importantly, each participant still had at least 14 nights of recordings.

Importantly, this research is the first to objectively measure sleep parameters in high-level para-athletes with multiple impairments. A breadth of knowledge has recently been gained relating to sleep in able-bodied athletes, however, the area of sleep in para-athletes is very limited. Therefore, this novel research may be used to build the foundation for this much needed area of sleep research.

## Practical Applications

Practitioners may be advised to track their athletes' sleep as inappropriate levels are evident from the current research. Attempting to understand risk factors to sleep is particularly important for those working in Para-sport as impairment related-challenges may play a role by influencing processes not limited to pain perception, thermoregulation, and sleep initiation/maintenance. Furthermore, specific strategies and appropriate planning may be needed to ensure sufficient sleep is attained during training camps. Finally, as there is limited information and understanding around sleep in high-level para-athletes, this study highlights the importance of continuing research in this area.

## Conclusions

In conclusion, highly trained WR athletes are on the borderline between moderate to poor quality sleep reflected by suboptimal sleep characteristics, such as prolonged WASO (>41 min) (Ohayon et al., 2017). Although objective values are suboptimal in and do not differ between athletes with a CSCI and NON-SCI, subjective ratings of sleep are worse in athletes with a CSCI. Furthermore, sleep is negatively influenced during periods of increased training or with early morning start times as seen during an in-season training camp. From a thermoregulatory perspective the difference in the T_core_ response between athletes with a CSCI and NON-SCI during the night-time period should be explored further.

## Data Availability Statement

The raw data supporting the conclusions of this article will be made available by the authors, without undue reservation.

## Ethics Statement

The studies involving human participants were reviewed and approved by Loughborough Univerisity Ethical Advisory Committee. The participants provided their written informed consent to participate in this study.

## Author Contributions

All authors listed have made a substantial, direct and intellectual contribution to the work, and approved it for publication.

## Conflict of Interest

The authors declare that the research was conducted in the absence of any commercial or financial relationships that could be construed as a potential conflict of interest.
